# Supplementary material for: Differences and commonalities in barriers and facilitators experienced by participants enrolled in an online behavioral weight management program: A qualitative comparison
Source: PLoS One. 2026 Feb 2;21(2):e0339411. doi: 10.1371/journal.pone.0339411 (PMC12863570; doi:10.1371/journal.pone.0339411)
Supplement: Attachment — Submitted filename: Response to Reviewers.docx [file pone.0339411.s002.docx]

Dear Editor and reviewers,

Thank you for your time to review this work and for the constructive comments. I have reviewed and updated the manuscript based on these suggestions and feel the manuscript is much improved. All changes have been tracked. I have addressed the editor comments first then the reviewer. I hope this layout is acceptable – I have also uploaded a tracked and clean version of the document. Please find details of this below.

Thank you again.

Yours Sincerely,

Dr Meigan Thomson

| **Editor/Journal Requirements** | **Response** |
| --- | --- |
| Please ensure that your manuscript meets PLOS ONE's style requirements, including those for file naming. | I have reviewed the supplied style templates and updated the formatting to match these. |
| Please provide an amended Funding Statement declaring this commercial affiliation, as well as a statement regarding the Role of Funders in your study.  Please also provide an updated Competing Interests Statement declaring this commercial affiliation along with any other relevant declarations relating to employment, consultancy, patents, products in development, or marketed products, etc. | As far as I can tell this was already clarified in these sections. JL at the time of the research was employed by Lancaster University but did receive personal fees from Novo Nordisk for consulting on advisory boards. JL now works full time with astra Zeneca (post-study). Neither of these had any influence over the data collection or analysis. Please let me know if I have missed anything |
| We noted in your submission details that a portion of your manuscript may have been presented or published elsewhere. Please clarify whether this publication was peer-reviewed and formally published. If this work was previously peer-reviewed and published, in the cover letter please provide the reason that this work does not constitute dual publication and should be included in the current manuscript. | This work was assessed as part of a PhD thesis. Work from this thesis regarding the impact of COVID has been published in Clinical Obesity. The content of this article has only been put forward for publication to PLOS ONE |
| We note that you have indicated that there are restrictions to data sharing for this study. For studies involving human research participant data or other sensitive data, we encourage authors to share de-identified or anonymized data. Please update your Data Availability statement in the submission form accordingly. | This statement already states that as part of the consent participants only agreed for the data to be shared for academic research and teaching hence the restriction. I am unclear what else I need to add. |
| Please remove all personal information, ensure that the data shared are in accordance with participant consent, and re-upload a fully anonymized data set. | The data in the article are de-identified. I am unclear what is needed. |
| Please review your reference list to ensure that it is complete and correct. If you have cited papers that have been retracted, please include the rationale for doing so in the manuscript text, or remove these references and replace them with relevant current references. Any changes to the reference list should be mentioned in the rebuttal letter that accompanies your revised manuscript. If you need to cite a retracted article, indicate the article’s retracted status in the References list and also include a citation and full reference for the retraction notice. | Thank you, I have reviewed the reference list and to the best of my knowledge it is correct. |

| **Reviewer 1 comments** | **Response** |
| --- | --- |
| Abstract -  a. Kindly include the rationale for using the socio-ecological model in the Abstract. | I have revised the abstract to meet the 300 word limit and be clearer on why the social ecological approach is suited |
| Introduction  a. Should be better organized and paragraphs restructured to present the arguments more coherently. For example, a clearer background and contexts is needed on obesity and its health consequences, role and impact of weight management programmes (WMPs), including BWMPs as well as evidence of success of WMPs compared to BWMPs, which the authors alluded to. Then, the theoretical framework, SEM, need to be clearer; perhaps by clearly identifying the multi-level factors influencing participation in WMPs. For example, in lines 69-80, the example of Boehmer et al given in lines 96-100 could make what constitutes “changes in environment” clearer. Similarly, “changes to social interactions” should be clarified. Also, the phrase “individuals who achieve a ≥5% weight loss” need better emphasis on the significance of that measure and possibly the time frame to achieving it. Although the authors addressed this in methods, I consider it is more appropriate in Introduction where the cutoff was introduced. | Thank you. I have updated the section and added in your suggestions regarding additional background on health, BWMPs and the SEM. I hope this strengthens the introduction and improves clarity. |
| Introduction  c. Also the authors stated as follows “Additionally, data collection typically occurs at the program’s conclusion or during a follow-up phase, potentially overlooking essential nuances and meaningful barriers and facilitators experienced during participation.” This does not seem to have been addressed as a finding of this study. I consider that the aspect should be deleted from the Introduction. | Thank you. I agree this is not highlighted in the discussion section. I think this is a really important unique aspect of the study so I have added this at the start of the discussion: Importantly, it captures these factors during program participation rather than relying on follow-up data collection, which can be affected by hindsight bias. This approach strengthens the study by offering real-time insights into the challenges participants face and a more nuanced understanding of the factors influencing weight management as they occur. |
| Methods  3. Methods: Although this section mostly covers the necessary details, I somehow found some part of the section challenging. I believe the current sequencing of information in some parts may be confusing to the readers. Therefore, reorganising the content to reflect the heading or subheadings is important. a. For example, it is unclear whether the wider mixed-methods project is the Second Nature online BWMP or that a mixed-methods study was conducted to evaluate the Second Nature online BWMP. Please make this clear under Study design and mention the overall study and its objectives to provide context for the qualitative component within the mixed methods design. Although I am uncertain how the Second Nature online BWMP programme fit with the mixed-methods project, I believe it should also be reported under the Study design. I suggest that the authors move ethical consideration details to a separate section because it is reported here and also partly duplicated in Recruitment (I seem to consider “Participants who completed the interview received £20 in form of a shopping voucher as compensation for their time and contribution to the research” an ethical issue than a Recruitment issue); Data collection (“Consent from participants was obtained either through email correspondence or recorded at the start of each interview. ……..audio files were uploaded and stored securely”). b. The Recruitment section could be changed to “Participant selection and sampling” or a similar broader heading. Although the section has relevant information, it requires more regarding what was the basis for the sample size of 40-50? Is this based on a similar previous study or arbitrary? What type of sampling technique was used, convenient, purposeful, snowballing? Also, some details here would be more appropriate in other sections. For example, I consider “Interviews were conducted during weeks 4 to 8 of the 12-week program.” to be more appropriate in Data collection section where it is already; therefore, please delete. Also “ To prevent bias, coding was completed before the research team accessed participants' weight loss outcomes. Once coding was finalised, each participant's weight loss status (i.e., whether they achieved a 5% reduction) was added to their transcript to enable comparative analysis.” may be more appropriate in data analysis, where it is already, or in strength and limitations. | Thank you.  I have edited and restructured a bit which hopefully makes the section clearer.  I have updated the details on the wider study and added a Ethical considerations subsection above the reflexivity section. I have kept the information about incentives in the same place as this is detailing the process. The sampling strategy is described in the Recruitment section (i.e., participants who signed up to second nature within 2 weeks of the study period were sent an invitation email, those who were interested contacted the research to participate. This is a cross between purposeful and convenience (i.e., only inviting new participants of the program from the paid arm – but inviting them all)  Regarding the target sample size, success rates in these programs vary so widely. Since we were blind until after coding the data and the purpose of the interviews was to compare those who did and those who did not reach a target of 5% we set a high target with the hopes of having good representation of both groups. I have updated the section as follows: Since it was uncertain how many participants would reach a ≥5% weight lost, a target sample size of 40–50 participants was set to ensure sufficient representation in both groups—those who met the 5% target and those who did not. Previous research indicates those meeting these targets are variable but typically just under half of participants will reach this level of weight loss (10), so the sample size was chosen to account for variability and to capture experiences from both outcomes.  I agree with the notes on duplication/placement of materials and have removed these accordingly. |
| Discussion  Consider briefly clarifying about the influence of other unexplored issues e.g. existing health conditions or comorbidities, around how facilitators and barriers may interact or differ by subgroups for added depth. | Thank you for this consideration, I have updated the following paragraph: Moreover, the data mainly focuses on barriers and facilitators encountered by female participants with higher income and education levels. It is probable that male or non-binary individuals, as well as those with lower incomes orless formal education may experience distinct barriers and facilitators across various social ecological domains. It is also common for people living with obesity to experience a comorbid health condition, which could interact with how barriers and facilitators manifest. For example, a comorbid condition could affect motivation, access to resources, or the perceived cost-benefit of behavior change (74). Additionally, examining obstacles related to resource availability and environmental infrastructure could provide valuable insights into the challenges faced by individuals in deprived areas and communities (75). |
| Overall Comments  Abstract: Correct punctuations errors: line 18 Grammar: lines 27-29;  Introduction Incomplete: lines 78-81  Methods Grammar: lines 244-246  Results; Lacking clarity/grammar: lines 265-266  Conclusion: Grammar: lines 700-702  Repetitions E.g.: lines 187 (Data collection) and lines 255-256 (Results)  Consistency: Behavioral change vs behavior change Tables should themes be fully capitalised vs not capitalised? E.g. Group Relations; Physiological response | Thank you for these observations. I have made the following corrections.  Line 18 - replacement of the comma  Line 27-29 - rephrased to: Both groups highlighted willpower, knowledge, social support, and perceived safety in their local area as key facilitators, noting that their absence acted as barriers. The presence of food at home and the workplace was additionally reported as a barrier due to temptation.  Lines 78-81 – I have rephrased this section: Given that participation in BWMPs is integrated into individuals' daily lives, it's essential to consider how various social-ecological factors—ranging from personal to environmental—shape the adoption of weight-related behavior changes.  Lines 244-246 – I have revised this section and surrounding sentences as these lines referred to different sections in the clean/tracked versions so I was unclear which section you were referring to: These representatives provided feedback on the clarity of the questions and made suggestions for improvements. They recommended including questions on social norms and support within the household and workplace regarding dietary behaviors, as well as more detailed inquiries about their weight loss history. These suggestions were incorporated into the interview schedule. Furthermore, to reduce biases in data coding and interpretation, 10% of the transcripts were independently coded by SAS and AM. In addition, MT remained blinded to participant outcomes during coding of all of the transcripts allowing for an unbiased comparison of themes between the groups. These measures strengthened the study's credibility and reduced potential biases throughout the research process.  Lines 700-702. I have revised this section: These findings highlight areas that programs can target to better support participants, particularly those likely to achieve less than a 5% weight loss. Key areas for considerations for programs include helping participants manage reactions to barriers, addressing social conflict and support , and providing tailored advice to where the program is situated  Repetitions – I have removed the duration of the interviews from the results section  Consistency – I’ve updated table 2 themes so they are capitalised. Behavior change vs behavioral change are interchangeable terms |
|  | |
| **Reviewer 2 Comments** | **Response** |
| • What is the average duration of such programs, or what is the minimum length of participation considered as completion? | Thank you for this comment. I have not added this into the introduction as the duration of these programs vary so widely. Average duration of such programs vary but a typical goal in weight management is to lose 5% of your baseline weight by 12 weeks. Second Nature is a 12 week program (described in the methods section under recruitment) |
| • What are the exit criteria for these programs? | Thank you for this comment. I have not added this into the introduction because programs do not typically have exit criteria. A few programs in the US provided via the workplace have targets that employees need to reach but this isn’t the norm. There is no exit criteria for Second Nature. Participants from the UK are either given a 12 week block from their GP or pay for a 12 week block (the participants in this study were all paying). Once the 12 weeks ends they can chose to restart the program or not pay for another block. The material covered in each block is the same and the participants retain access to the materials and recipes. The other way they may exit the program is by dropping out (i.e., stop engaging/using the program) |
| (Line 262) — Please justify describing only the female respondents in the descriptive table. It would be helpful to clarify whether this focus was based on sample composition, analytic relevance, or another reason. | Thank you for your query.  The text and table do not refer exclusively to female participants. As indicated in the gender section, there were 48 participants in total (as shown in the column heading), of whom 40 were female and 8 were male. The table has been left unchanged, as this is a standard approach to reporting demographic data.  Both the table and the results section present data from the entire sample. All participants identified as either male or female, which is why no further breakdown of gender identity was provided. |
| Limitations: (Lines 686–691) — The points raised in this section appear to align more closely with recall bias and the absence of data triangulation. You may wish to reframe them accordingly. | Thank you, this point does not reflect to problems with data triangulation or recall bias (which is likely an issue when asking participants about factors impacting their weight loss during a program). The point here is that the participants were interviewed midway through the program so any later barriers/facilitators will not have occurred yet and participants may have focused on current barriers/facilitators rather than recalling all barriers/facilitators up to that point. It is likely barriers/facilitators change throughout the course of the weight management programs depending on contextual influences and how well they are doing in the program so it would be useful to do short interviews regularly throughout a program to determine whether these do change and if they link to certain weight loss trajectories so support could be in place where feasible. I hope this clarifies what we meant. I have updated the section as follows:  Additionally, it is important to note that the data were collected at a single timepoint during the early to mid-stages of the program which may have prevented the identification of barriers and facilitators that arise later in the program. Such factors are likely to evolve over time depending on individuals’ weight-loss experiences. To better understand how barriers and facilitators change and how they relate to different weight-loss trajectories, future research should incorporate interviews at multiple timepoints |
| Please review the placement of the comma on line 18. | Thank you, this has been moved as follows: Online behavioral weight management programs offer a scalable solution to combat overweight and obesity, yet often face challenges with high dropout and varying success rates. |
| Lines 80–81: Please check and revise these sentences for grammatical accuracy and clarity. | Thank you for indicating that reframing was needed. I was uncertain whether your comment pertained solely to the sentence spanning these lines or also to the adjacent sentences, so I have revised the section accordingly:  Given that participation in BWMPs is integrated into individuals’ daily lives, it is essential to consider how various social–ecological factors—from personal to environmental—shape the adoption of weight-related behavior changes. Despite BWMPs typically being implemented within community settings, much of the research on barriers and facilitators to weight management has focused primarily on individual-level factors, with only limited exploration of interpersonal influences. |
| Lines 92–95: Please review and correct these sentences to ensure coherence and precision. | Thank you for indicating that reframing was needed. I was uncertain whether your comment pertained solely to the sentence spanning these lines or also to the adjacent sentences, so I have revised the section accordingly:  Studies have predominantly shown that individuals who achieve ≥5% weight loss tend to report higher levels of motivation, greater perceived control over their weight and related behaviors, increased self-efficacy, and stronger social support (22–28). Research on environmental influences underscores the significance of food availability, access to food and exercise facilities, the presence of green space, and the visibility of convenience foods in shaping obesity prevalence and weight-related behaviors (29–32). Perceptions of the environment also influence weight-related behaviors. For example, Boehmer and colleagues found significant associations between obesity prevalence and perceptions that an area was predominantly residential, lacked points of interest (e.g., leisure facilities or green space), and had fewer or poorly maintained sidewalks (33). |
